# Prospective Follow-up of Children with Idiopathic Growth Hormone Deficiency After Termination of Growth Hormone Treatment: Is There Really Need for Treatment at Transition to Adulthood?

**DOI:** 10.4274/jcrpe.0010

**Published:** 2018-07-31

**Authors:** Emine Çamtosun, Zeynep Şıklar, Merih Berberoğlu

**Affiliations:** 1 Ankara University Faculty of Medicine, Department of Pediatric Endocrinology, Ankara, Turkey

**Keywords:** Growth hormone deficiency, transition, childhood

## Abstract

**Objectives:**

Continuation of growth hormone (GH) treatment in adolescents with severe childhood onset idiopathic GH deficiency (IGHD) during the transition period, irrespective of achievement of final height, is still debatable. We aimed to prospectively investigate the metabolic profile, bone mineral density (BMD) and body composition of patients with IGHD in whom GH treatments were terminated after they had reached their final height, six months after the cessation of therapy.

**Methods:**

Twelve patients, six of whom had peak GH levels <5 ng/mL [permanent GH deficiency (GHD), group 1], and six who had peak GH levels >5 ng/mL (transient GHD, group 2) after insulin stimulation test were evaluated for anthropometric and laboratory parameters including fasting blood glucose (FBG), fasting insulin, lipid profile, BMD, body composition measurements and 24-hour ambulatory blood pressure monitoring before (baseline) and at six months after discontinuation of GH.

**Results:**

No differences were found in clinical, laboratory, BMD and body composition measures between groups 1 and 2 at baseline. All IGHD patients had significant increments of body weight (BW), body mass index (BMI), BMD, total body fat (TBF), TBF%, truncal fat (TF) and TF% after GH cessation. Six months later BW, BMI, BMD and TF% was increased significantly while FBG and lipids showed no change in group 1. In group 2, TBF% and TF% were increased, FBG, total cholesterol and high-density lipoprotein decreased after six months. Changes in these parameters in group 2 were not statistically different from group1.

**Conclusion:**

TF% increase in both groups after cessation of therapy. We did not observe a clinical condition requiring GH treatment in any of the study subjects during the follow-up period.

## What is already known on this topic?

Continuation of growth hormone (GH) treatment in adolescents with severe, childhood-onset idiopathic GH deficiency during the period of transition to adult care is still debatable.

## What this study adds?

We did not observe a clinical condition requiring growth hormone (GH) treatment in any of the study subjects, half of whom had idiopathic GH deficiency and half with transient growth hormone deficiency, during the six months subsequent to cessation of treatment.

## Introduction

Growth hormone (GH) treatment is generally applied to stimulate longitudinal skeletal growth in children with idiopathic GH deficiency (IGHD) and is terminated when final height is attained and epiphyseal closure has occurred. However, it has been reported that body mass (i.e. muscle and bone mass) of adult patients with severe childhood onset GHD who had been treated with GH until they achieved their final height, was significantly less than the body mass of young adults with adult onset GHD (1). Somatic development, including body composition, muscle mass maturation and skeletal mineralization is completed during the transition from late adolescence to early adulthood and GH is believed to play a role in this process (2).

GHD adults receiving hormone replacement therapy were reported to have increased body fat, insulin resistance and dyslipidemia with low-density lipoprotein (LDL), high serum triglycerides (TG), and high-density lipoprotein (HDL) levels. Increased prevalence of hypertension, premature atherosclerosis, mortality from cardiovascular diseases and decreased quality of life are also observed in these patients (3). These observations suggest that GH may also play a role in the prevention of metabolic and cardiovascular diseases.

Initial small-scale trials reported between 1991-1994 have demonstrated a reduction in muscle mass and an increase in fat mass during the 3-12 months transition period after termination of GH therapy (3). Further studies evaluated the effects of discontinuation of GH on metabolic profile in the transition period. Some of these studies evaluated the effects of GH therapy on bone mineralization (4) and some on body composition (5) using different study protocols. These studies concluded that GH was necessary for better adult metabolic profile, peak bone mass and body composition in GH deficient adolescents after they had attained their final height. In contrast, a more recent study concluded that GH deficient patients properly treated during childhood could have normal bone mineral density (BMD), body composition, cardiac function, muscle strength, carbohydrate metabolism, lipid metabolism and a good quality of life when they reached their adult height (6). These authors also reported that continuation of GH therapy for an additional two years did not change any of these parameters when compared to placebo-treated or control subjects. Continuation of GH treatment in adolescents with severe GHD during the transition period, irrespective of achievement of final height, is therefore still debatable.

In this study, we aimed to investigate prospectively, six months after the cessation of therapy, the metabolic profile, BMD and body composition of patients with isolated childhood onset GHD in whom GH treatment was terminated after they reached their final height.

## Methods

This was a single center, prospective clinical study carried out in accordance with good clinical practice guidelines, with appropriate ethical approval and signed informed consent. Ethical approval was given by Ankara University Ethical Committee (approval number: 06-240-13).

We evaluated insulin tolerance tests (ITT) in a group of patients with childhood onset IGHD who were followed at Ankara University, Department of Pediatric Endocrinology, who had received GH treatment during their childhood and had been off GH when they reached their final height. Twelve patients who gave consent were included in the study and followed-up prospectively.

All of these twelve patients conformed to the clinical and diagnostic criteria for isolated IGHD and had GH peaks less than 10 ng/mL following two different provocative pharmacological stimuli (levodopa and ITT) prior to beginning treatment. None of these patients had any other pituitary hormone deficiencies. All patients had received subcutaneous recombinant human GH treatment at a dose of 0.2 mg/kg/week, over six days out of seven each week until they reached their final height. Final height achievement was defined as attainment of bone age (BA) ≥14 years for girls, ≥16 years for boys, closure of epiphyseal plaques and a decreased height velocity <2 cm/year (7). Diagnosis of organic GHD, GH neurosecretory dysfunction, multiple anterior pituitary hormone deficiencies and requirement for any additional treatment for a chronic disease or a complex syndrome constituted the criteria for exclusion. 

**Figure 1:**
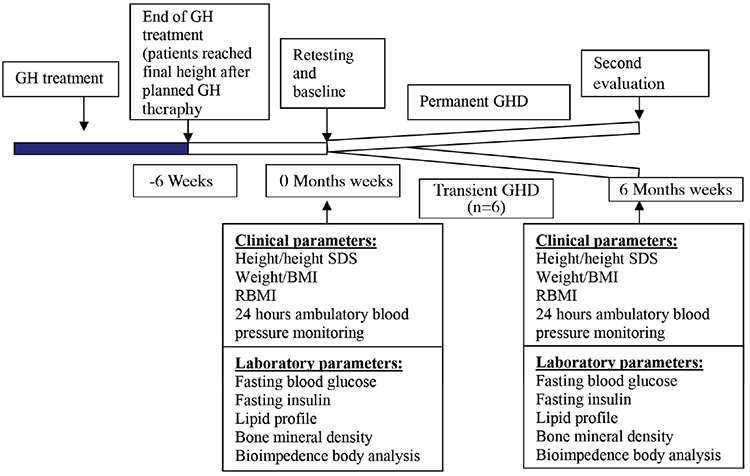
Study design GH: growth hormone, GHD: growth hormone deficiency, SD: standard deviation, SDS: standard deviation score, BMI: body mass index, RBMI: relatif body mass index

The study design is given in Figure 1. Twelve patients who achieved their final height were prospectively followed-up. They underwent basal anthropometric and laboratory evaluation including measurement of body weight (BW), calculation of body mass index (BMI), calculation of BMI for height percentage (BMI %), measurement of serum insulin like growth factor 1 (IGF-1) level, estimation of IGF-1 standard deviation score (SDS), measurement of IGF binding protein 3 (IGFBP-3) and estimation of IGFBP-3 concentration SDS, measurement of fasting blood glucose (FBG), fasting insulin (FI), fasting total cholesterol (TC), LDL-cholesterol (C), HDL-C, TG levels just before the cessation of GH therapy while they were still being administered GH. Besides, a 24-hour ambulatory blood pressure monitoring and a dual energy X-ray absorptiometry scan to asses BMD were performed in each patient. Z-scores for BMD, both for chronological age (CA) and BA were calculated according to BMD reference data for healthy Turkish children (8). Blood pressure monitoring was interpreted as systolic/diastolic overload, which was defined as the percentage of high blood pressure measurements among all systolic/diastolic measurements for night and day separately. Body composition measurements [total body fat (TBF) mass, TBF%, truncal fat (TF) mass, TF%, total body muscle (TBM) mass and TBM%] were performed via TANITA Body Composition Analyzer, BC-418 MA III. 

GH therapy was discontinued after the baseline evaluations in all subjects and six weeks later each patient was re-evaluated for GHD by ITT and IGF-1, IGFBP-3 levels. A peak serum GH level below 5 ng/mL was defined as GHD (9). Peak GH response <5 ng/mL was defined as permanent and peak GH response >5 ng/mL was defined as transient GHD. Patients were grouped as permanent (group 1, n=6) and transient (group 2, n=6) GHD, according to their peak GH response.

Six months after discontinuation of GH treatment anthropometric and laboratory evaluations similar to baseline evaluations were repeated in each patient. These parameters and baseline parameters in all patients were compared both within each group and between group 1 (permanent GHD) and group 2 (transient GHD) (9).

Serum IGF-1, IGFBP-3, fasting insulin and growth hormone levels were measured using the radioimmunoassay method by gamma counter analyser using DSL kit. SDS were calculated for IGF-1, IGFBP-3 according to the reference data of the analyser. TC, HDL-C, TG and fasting glucose were measured by the enzymatic method using the “Beckman Coulter Unicel DxC 800” analyser. LDL-C was derived from other lipid measurements.Atherogenesis index (AI) was calculated as TC/HDL-C (10). The homeostasis model assessment (HOMA) was used to evaluate insulin resistance and HOMA was calculated using the following formula: HOMA=[fasting insulin (µU/mL) X glucose (mg/dL)] /22,5x18 (11). Serum cortisol (for insulin tolerance test) was assessed by immunoenzymatic method with the Roche E170 analyser.

### Statistical Analysis

The data were analyzed using the SPSS software program for Windows, version 16.0 (SPSS, Inc., Chicago, IL, USA). Continuous variables were compared between groups using Mann-Whitney U test. For the repeated measures, we also used Mc Nemar or Wilcoxon matched-pair signed-rank test. The values for all parameters except for gender and blood pressure non-dipping ratio were expressed as mean ± SDS (median). P<0.05 was considered as statistically significant.

## Results

Patients who had received GH treatment during their childhood for IGHD and had been off GH treatment when they reached their final height were retested with ITT in our pediatric endocrinology department and among them 12 patients who agreed to participate in the study were followed-up prospectively.

All 12 patients demonstrated a significant increase in BW (p=0.033) but not in BMI and BMI% six months after GH cessation when compared to baseline. IGF-1 and IGF-1 SDS decreased significantly (p=0.006 and p=0.007, respectively). FBG and HDL-C also decreased, while FI and other parameters of their lipid profiles did not change. Although BMD increased (p=0.01), BMD z-score for CA and BA were not different after 6 months. TBF mass, TBF%, TF mass and TF% increased significantly (p values respectively 0.008; 0.028; 0.012; and 0.005). TBM mass decreased but TBM% did not change. There were no significant changes with respect to blood pressure measurements.

### Clinical and Laboratory Characteristics of Group 1 Patients at Baseline and Six Months Later

In group1 BW and BMI increased significantly after six months from discontinuation of GH (p=0.043 and p=0.043, respectively) however BMI% did not change (Table 1A). IGF-1 and IGF-1 SDS significantly decreased (p=0.028 and p=0.028, respectively) and changes in IGFBP-3 and IGFBP-3 SDS were not statistically significant. FBG, FI, lipid profile and AI showed no change. BMD increased significantly (p=0.043), but BMD z-scores for CA and BA did not. TBF mass and TF% increased, while TBF%, TF mass, TBM mass and TBM% did not change significantly (p=0.043) (Table 1B).

### Clinical and Laboratory Characteristics of Group 2 Patients, Baseline and Six Months Later

BW, BMI and BMI% did not change significantly from baseline in group 2 (Table 2A). IGF-1, IGF-1 SD, IGFBP-3 and IGFBP-3 SD were not statistically different six months after discontinuation of GH therapy. FBG, TC and HDL-C decreased by six months after cessation of treatment (p values respectively 0.043; 0.046 and 0.046). FI, LDL-C, TG and AI, as well as BMD, BMD z-scores for CA and BA were statistically similar at baseline and at the six-month follow-up (Table 2B). TF% increased significantly in this group, but other parameters of body composition did not change (Table 2B).

### Differences Between Groups 1 and 2

At baseline and after six months, group 1 and group 2 were similar with respect to all study parameters.

## Discussion

It has become clear that GH and GH therapy affect more than just linear growth and have additional effect on metabolism, bone maturation and body composition. Therefore, the consequences of GH discontinuation in childhood onset GHD, after achieving final height, have become a concern among endocrinologists. There are contradictory results concerning the effect of GH on various biochemical and anthropometric parameters after discontinuation of GH in the literature. Several studies have demonstrated metabolic changes, while some others have reported no change. Thus, the clinical implication of these changes, if there are any, is debatable.

We performed this prospective study in order to evaluate the need for continuation of GH during the transition period in this group of patients. There are only a small number of studies on this topic. Some of them have evaluated effects of GH discontinuation on a specific field such as metabolic profile, or bone mass, or body composition. Most of these studies are multicenter and are made up of a heterogeneous group of subjects which include GHD patients with different etiologies (3,4,5,12).

In our study, permanent GHD subjects (group 1) had significantly increased BW and BMI at six months after discontinuation of GH however their BMI% did not change at all. In addition, the increase in BW and BMI in group 1 was not different from the increase observed in the transient GHD group, which is consistent with the literature (3,6). Johannsson et al (3) have evaluated 40 GHD patients (including organic GHD and multiple anterior pituitary hormone deficiencies) in 21 GH deficient, 19 GH sufficient and 16 healthy controls annually, after discontinuing GH treatment for a follow-up period of two years. They have reported increased BMI in both the transient GHD and control groups, however BMI did not differ between the groups. Also, the BMI did not change in their GH deficient group over the follow-up period. Mauras et al (6) studied 58 childhood onset GHD with heterogeneous etiology after GH discontinuation. According to retest, 40 of their patients showed transient GHD while18 of their patients were GH sufficient. Twenty-five patients with transient GHD were treated with GH, 15 treated with placebo. No significant differences were found with respect to weight and BMI over two years among their study population. It is well known that GH itself has a lipolytic effect. Increase in BW in the permanent GHD group, and TF% both in the permanent and the transient group after cessation of GH therapy may be related to reduction of the GH dependent lipolytic effect.

IGF-1 and IGF-1 SDS significantly decreased after discontinuation of therapy taking all twelve patients as a whole while these values did not differ in the GH sufficient group. IGFBP-3 and IGFBP-3 SD did not change statistically in either group. Significant decrease in IGF-1 level in the permanent GHD group after discontinuation of GH is consistent with the literature (3,6,13). Decrease in IGF-1 level in group 1 can be a representative factor for persisting GH deficiency. Probably due to the small sample size, no difference with respect to IGF-1 was observed between baseline and after six months of GH cessation in group 2.

We also evaluated the metabolic effects in our study population. FBG, FI and lipid profile parameters were all similar compared to baseline values after six months in the permanent GHD group. In group 2 decreases in FBG, TC, HDL-C after six months were found but their FI, LDL-C, TG and AI values did not change. All parameters were within the normal ranges. Decrease in FBG in the transient group after six months, although not observed in group 1, may be due to the weaning effect of GH on glucose metabolism. In our opinion, no significant negative effects on glucose metabolism were observed in either group at six months after discontinuation of GH. There are contradictory results related to these parameters in the literature. Johannsson et al (3) in their study of 21 GHD, 19 GHS patients and 16 healthy controls reported that TC, LDL-C and apolipoprotein B were all higher in the permanent GHD group than the other two groups before GH discontinuation. TC and LDL-C increased in both GH deficient and GH sufficient groups but not in healthy controls at two years after discontinuation of GH. After two years the serum levels of these lipids were therefore still higher in the GH deficient group than the other groups (3). HDL-C increased in healthy controls after two years. The permanent GHD group had statistically lower HDL-C compared to the GH sufficient group after 2 years (3). In the study of Mauras et al (6) there was no significant difference with respect to either FG or homeostatic model assessment insulin resistance in GH deficient (n=40; 25 received further GH and 15 placebo) and sufficient (n=18) groups at baseline or throughout the 24-month study period. Their study also demonstrated no significant difference across the groups with respect to TC, LDL-C, HDL-C, TC/HDL ratio and TG at baseline, at baseline vs. month 12 and at baseline vs. month 24 (6). Carroll et al (13) reported similar lipoprotein profiles in GH continuation and discontinuation groups at 12 months. 

We observed that BMD increased significantly in GH deficient subjects at six months after discontinuation of GH therapy whereas BMD Z-scores for CA and BA did not change. Group 2 demonstrated no change in BMD and BMD z-scores for CA and BA after six months. Limited previous studies have reported an increase in BMD of GH deficient patients after discontinuation of GH treatment at final height (4,12). Fors et al (12) reported similar BMD values at baseline and after two years among three groups (40 childhood onset GHD retested of whom 21 were GH deficient and 19 GH sufficient in comparison with 16 healthy controls), after discontinuation of GH therapy at or near final height. They concluded that bone mass measured by DXA continued to rise for two years, but bone markers showed that bone formation decreased during the same period. This study does not rule out the possibility of slow long-term loss of bone in adults with GHD (12). Some studies showed bone loss in longitudinal measurements in the same population (4,13,14,15). Kaufman et al (14) showed that in childhood-onset GHD in a group of full-grown men treated with GH, they had low adult bone mass, despite prior GH substitution. In a large multi-center prospective study of 149 young adults with childhood-onset GHD who had completed linear growth and who were randomized to receive 12.5 µg/kg/d; 25 µg/kg/d or no treatment for two years the following was reported; over two years, total bone mineral content (BMC) and BMD were significantly increased in the control group, but increases in both treatment groups were greater than that of the controls and that there was no significant dose effect (4). The authors suggested that GH treatment after attainment of final height should not be discontinued for optimal progress to peak bone mass. However, in this study BMD z-scores were not evaluated and it was not shown whether the increase of BMD in the control group was enough for a normal BMD z-score. In our study, BMD z-scores did not decrease after six months in either group. Moreover, a statistically significant increase was detected in the permanent GHD group, this may be due to either small number of cases in this group, or the lasting effect of GH on bone metabolism. Baroncelli et al (15) showed in16 GHD adolescents that lumbar BMD area was <-2 SD in 70% and lumbar BMD volume was between 0 and -2 SD of normal at final height. They showed that after discontinuation of GH the timing of lumbar peak BMD area and volume were delayed in GHD patients and that lumbar BMD area and volume were increased until peak, then significantly decreased two years after peak compared with controls (15). 

We have demonstrated that TF% increased significantly but that TBF mass, TBF%, TF mass, TBM mass and TBM% did not change in the permanent GHD group six months after GH discontinuation. We have also shown that, TF% and TBF mass increased significantly but TBF%, TF mass, TBM mass and TBM% did not change in the transient GHD group. The amount of increase of TF% was not different in the transient GHD and permanent GHD groups. In an earlier study, Ogle et al (16) compared eight adolescent GHD patients (whose GH treatment had ceased) with seven age matched healthy subjects for 12 months. They observed that lean body mass (LBM) decreased significantly in patients with GHD at 12 months whereas there was a non-significant increase in LBM in the control group. The percentage of body fat increased in all patients with GHD at six and 12 months with no significant increase in the control group. The mean android (trunk)/gynoid (legs) fat ratio increased, though non-significantly, in patients with GHD at 12 months while no change was observed in the control group. Vahl et al (17) showed that TBF increased and resumption of GH increased LBM after discontinuation of GH for 1 year. 

Johannsson et al (3) evaluated 40 GHD patients annually, after discontinuing GH treatment for two years. They showed that the amount of total body and abdominal fat mass throughout the study and increase in these masses were more marked in permanent GHD patients than in the transient GHD and control subjects (3). Attanasio et al (5) randomized 149 GHD patients who received GH until final height as GH treated (58 pediatric dose, 59 adult dose) and not treated groups. GH treated patients gained a significant amount of LBM and lost significant fat mass compared to the no treatment group during two years. There was no dose effect (5). A multi-centered study reported similar results for one year, with respect to continuation or discontinuation GH treatment (18).

The strengths of our study are its prospective design and the simultaneous evaluation of many metabolic parameters. In addition, our study group consisted of only idiopathic isolated GHD patients. This enables us to rule out the effects of other hormone deficiencies, probable inappropriate/non-homogenous replacement therapies and the effects of other organic pathologies and their treatments such as chemotherapy or radiotherapy.

### Study Limitations

The limitations of our study are the short follow-up period and small sample size. Thus, in our opinion this study can be accepted as a well-designed pilot study which should be extended in terms of numbers of subjects and duration of follow-up for further and more robust information.

## Conclusion

BW, BMI and truncal adiposity increased after cessation of GH if GHD was permanent. However, these increases were not significant compared to the transient GHD group. Despite discontinuation of GH, the pubertal increase in BMD continued in GHD patients. Although FBG was not high during GH therapy, it decreased to a safer range after cessation of therapy while FI showed no change. Although we did not observe a clinical condition requiring GH treatment in any of the study subjects during the follow-up period, which coincided with the transition period, longer follow-up is needed to assess the need for treatment during the transition period. In our opinion, until sufficient evidence accumulates, patient follow-up in terms of the above-mentioned parameters and treatment only of patients with a clinical need, instead of routine continuation of GH during the transition period, might be a safe and feasible option.

## Figures and Tables

**Table 1A:**
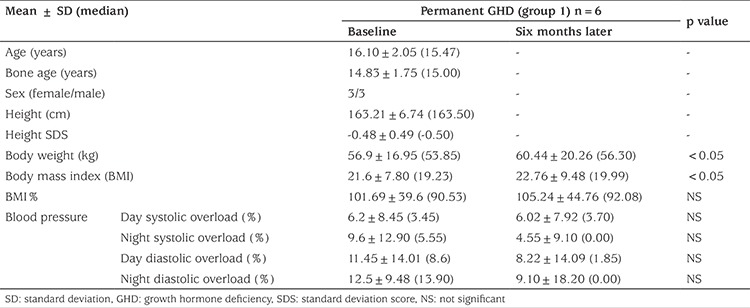
Clinical findings at baseline and after six months in the permanent growth hormone deficiency patients

**Table 1B:**
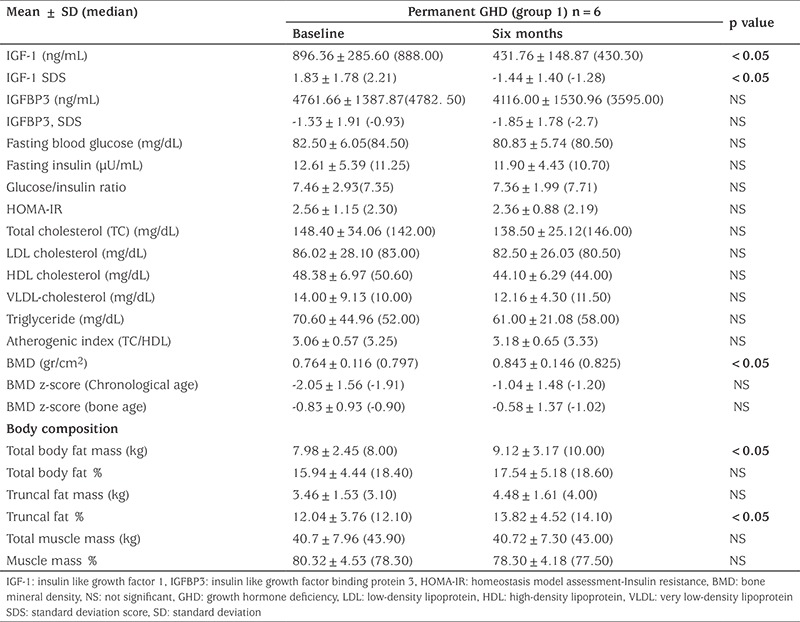
Laboratory findings in the permanent growth hormone deficiency (group 1) at baseline and after six months

**Table 2A:**
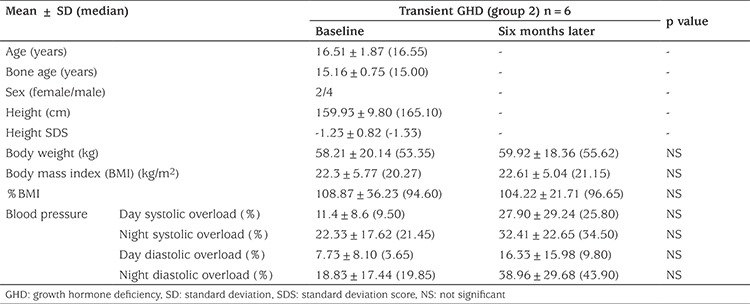
Clinical comparison of transient growth hormone deficiency (group 2) at baseline and after six months

**Table 2B:**
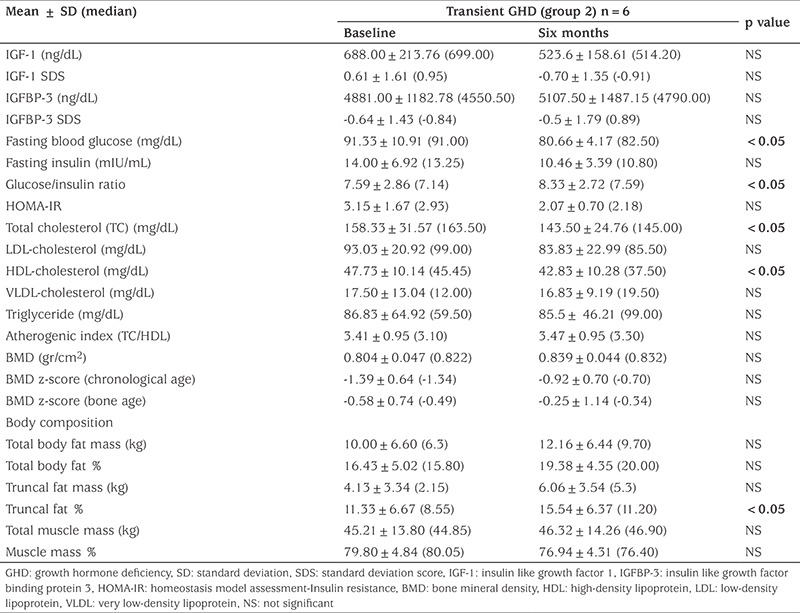
Comparison of laboratory findings at baseline and after six months in the transient growth hormone deficiency (group 2)

## References

[1] Attanasio AF, Howell S, Bates PC, Frewer P, Chipman J, Blum WF, Shalet SM (2002). Body composition, IGF-I and IGFBP-3 concentrations as outcome measures in severely GH-deficient (GHD) patients after childhood GH treatment: a comparison with adult onset GHD patients. J Clin Endocrinol Metab.

[2] Chamberlain SH, Levy RA (2012). Transition care of patients with growth hormone deficiency from pediatric endocrinologists to adult endocrinologists. Endocr Pract.

[3] Johannsson G, Albertsson-Wikland K, Bengtsson BA (1999). Discontinuation of growth hormone (GH) treatment: metabolic effects in GH-deficient and GH-sufficient adolescent patients compared with control subjects. Swedish Study Group for Growth Hormone Treatment in Children. J Clin Endocrinol Metab.

[4] Shalet SM, Shavrikova E, Cromer M, Child CJ, Keller E, Zapletalová J, Moshang T, Blum WF, Chipman JJ, Quigley CA, Attanasio AF (2003). Effect of growth hormone (GH) treatment on bone in postpubertal GH-deficient patients: a 2-year randomized, controlled, dose-ranging study. J Clin Endocrinol Metab.

[5] Attanasio AF, Shavrikova E, Blum WF, Cromer M, Child CJ, Paskova M, Lebl J, Chipman JJ, Shalet SM;, Hypopituitary Developmental Outcome Study Group (2004). Continued growth hormone (GH) treatment after final height is necessary to complete somatic development in childhood-onset GH-deficient patients. J Clin Endocrinol Metab.

[6] Mauras N, Pescovitz OH, Allada V, Messig M, Wajnrajch MP, Lippe B;, Transition Study Group (2005). Limited efficacy of growth hormone (GH) during transition of GH-deficient patients from adolescence to adulthood: a phase III multicenter, double-blind, randomized two-year trial. J Clin Endocrinol Metab.

[7] Cohen P, Rogol AD, Deal CL, Saenger P, Reiter EO, Ross JL, Chernausek SD, Savage MO, Wit JM;, 2007 ISS Consensus Workshop participants (2008). Consensus statement on the diagnosis and treatment of children with idiopathic short stature: a summary of the Growth Hormone Research Society, the Lawson Wilkins Pediatric Endocrine Society, and the European Society for Paediatric Endocrinology Workshop. J Clin Endocrinol Metab.

[8] Goksen D, Darcan S, Coker M, Kose T (2006). Bone mineral density of healthy Turkish children and adolescents. J Clin Densitom.

[9] Thomas M, Massa G, Maes M, Beckers D, Craen M, François I, Heinrichs C, Bourguignon JP;, Belgian Study Group for Paediatric Endocrinology (BSGPE) (2003). Growth hormone (GH) secretion in patients with childhoodonset GH deficiency: retesting after one year of therapy and at final height. Horm Res.

[10] Malaspina JP, Bussiere H, Le G (1981). The total cholesterol/HDL cholesterol ratio: a suitable atherogenesis index. Atherosclerosis.

[11] Matthews DR, Hosker JP, Rudenski AS, Naylor BA, Treacher DF, Turner RC (1985). Homeostasis model assessment: insulin resistance and beta-cell function from fasting plasma glucose and insulin concentrations in man. Diabetologia.

[12] Fors H, Bjarnason R, Wirent L, Albertsson-Wikland K, Bosaeust L, Bengtsson BA, Johannsson G (2001). Currently used growth-promoting treatment of children results in normal bone mass and density. A prospective trial of discontinuing growth hormone treatment in adolescents. Clin Endocrinol (Oxf).

[13] Carroll PV, Drake WM, Maher KT, Metcalfe K, Shaw NJ, Dunger DB, Cheetham TD, Camacho-Hübner C, Savage MO, Monson JP (2004). Comparison of continuation or cessation of growth hormone (GH) therapy on body composition and metabolic status in adolescents with severe GH deficiency at completion of linear growth. J Clin Endocrinol Metab.

[14] Kaufman JM, Taelman P, Vermeulen A, Vandeweghe M (1992). Bone mineral status in growth hormone-deficient males with isolated and multiple pituitary deficiencies of childhood onset. J Clin Endocrinol Metab.

[15] Baroncelli GI, Bertelloni S, Sodini F, Saggese G (2004). Longitudinal changes of lumbar bone mineral density (BMD) in patients with GH deficiency after discontinuation of treatment at final height; timing and peak values for lumbar BMD. Clin Endocrinol (Oxf).

[16] Ogle GD, Moore B, Lu PW, Craighead A, Briody JN, Cowell CT (1994). Changes in body composition and bone density after discontinuation of growth hormone therapy in adolescence: an interim report. Acta Paediatr Suppl.

[17] Vahl N, Juul A, Jorgensen JO, Orskov H, Skakkebaek NE, Christiansen JS (2000). Continuation of growth hormone (GH) replacement in GH-deficient patients during transition from childhood to adulthood: a two-year placebo-controlled study. J Clin Endocrinol Metab.

[18] Monson JP (2005). Indications for GH replacement in adolescents and young adults. J Endocrinol Invest.

